# Primary displacement predicts complications and poorer outcomes after pediatric proximal radius fractures: A retrospective study of 140 fractures

**DOI:** 10.1177/18632521261434093

**Published:** 2026-03-25

**Authors:** Kaj Zilliacus, Yrjänä Nietosvaara, Ilkka Helenius, Reetta Kivisaari, Niko Kämppä, Petra Grahn

**Affiliations:** 1 Department of Pediatric Orthopedics and Traumatology, New Children’s Hospital, Helsinki University Hospital, University of Helsinki, Helsinki, Finland; 2 Department of Orthopedics and Traumatology, Bridge Hospital, Helsinki University Hospital, University of Helsinki, Helsinki, Finland; 3 Department of Pediatric Surgery, Kuopio University Hospital, University of Eastern Finland, Kuopio, Finland

**Keywords:** Pediatric proximal radius fracture, pediatric radial neck fracture, complications, long-term outcome, pediatric elbow fracture

## Abstract

**Purpose:**

Pediatric proximal radius fractures frequently lead to complications and unsatisfactory results, yet the medium- to long-term outcomes of these injuries remain insufficiently studied.

**Methods:**

We treated 140 proximal radius fractures in 138 children (median age 10, range 1–16 years) between 2014 and 2019. Fracture characteristics, treatment, complications, patient-reported, and functional outcomes were assessed at a median follow-up of 6.8 years (range 5–10 years) through clinical assessment or telephone interview in 110 patients (80%).

**Results:**

Less severe fractures (Judet types I–II) were most common (84/140), while 40% were Judet types III–IV. Most fractures (66%) were treated nonoperatively. Complications occurred in 25% of cases and were mainly associated with displacement ≥3 mm (Odds ratio (OR) 6.7, 95% confidence interval (CI) 2.7–19.1), physeal involvement (OR 5.1, 95% CI 2.0–15.7), and higher Judet classification (OR 4.2, 95% CI 1.9–9.7). Unfavorable functional outcomes occurred in 16% (11/67) and were more frequent after surgical treatment (OR 5.0, 95% CI 1.3–19.6) and in patients with complications (OR 9.2, 95% CI 1.8–47.0).

**Conclusion:**

In pediatric proximal radius fractures, primary displacement ≥3 mm, higher Judet’s class, and physeal involvement increased the risk of complications and unfavorable long-term outcomes.

**Level of evidence:**

Prognostic study, Level III

## Introduction

Proximal radius fractures, involving the radial neck or head, account for 1% of all pediatric fractures and up to 16% of pediatric elbow injuries,^1–5^ with the peak of incidence at 8–10 years of age.^1,4,6,7^ Most of these fractures are extra-articular and involve the proximal metaphysis of the radius.^4,8^ Fractures with <30° of angulation and <3 mm of displacement are believed to do well with immobilization in situ, whereas >30° of angulation or >3 mm of displacement is often considered an indication for intervention.^2,5,6^ Pediatric proximal radius fractures have a high complication rate, and poor outcomes have been reported in up to one-third of the cases.^1,5,7,9^ Age >10 years, increased fracture severity, and open reduction have been claimed to predict complications and poor functional outcomes.^1,7^ However, there are no previous reports on long-term functional outcomes, including patient-reported outcome measures (PROMs).^10^

The aim of this study was to evaluate outcomes of pediatric proximal radius fractures, including PROMs, after a minimum 5-year follow-up and to identify factors predicting complications and poorer results. We hypothesized that more severe proximal radius fractures requiring open reduction would provide poor long-term functional outcomes as compared to closed management.

## Materials and methods

### Design and data source

This study was conducted at the Helsinki University Hospital and is based on a single-center registry-based cohort at a level 1 pediatric trauma center serving a catchment population of 1.7 million. STROBE guidelines were followed.

### Parameters

**Figure 1.**
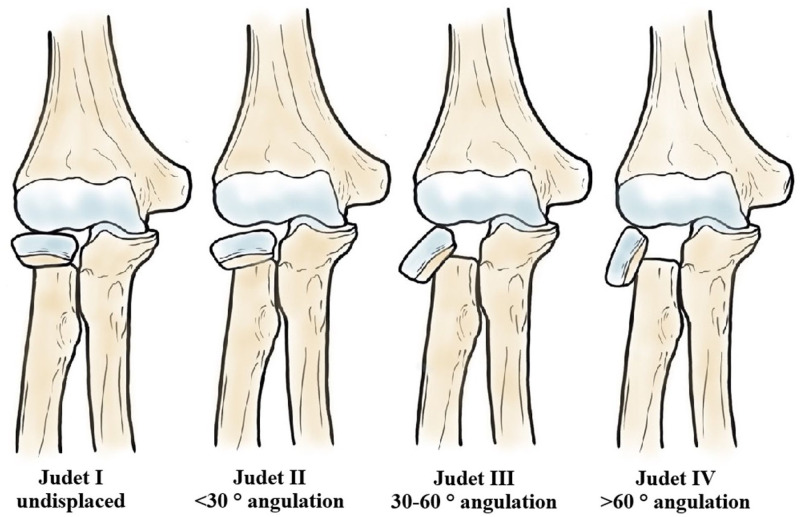
Fracture severity was classified according to Judet’s classification.^11^

**Figure 2.**
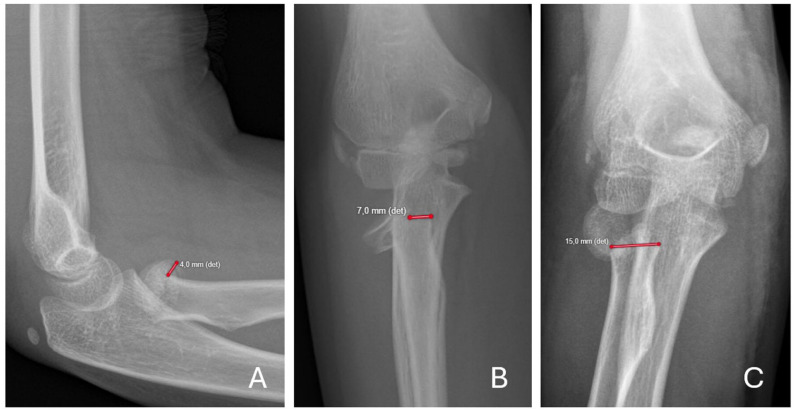
Maximal displacement was measured on both AP and lateral radiographs, and the greater value was recorded. Examples: (a) Judet type II fracture with 4 mm maximal displacement on the lateral view; (b) Judet type III fracture with 7 mm maximal displacement on the AP view; and (c) Judet type IV fracture with 15 mm displacement on the AP view. det: detector; AP: Anteroposterior; distance was measured relative to the detector positioned beneath the arm.

All 0- to 15-year-old children treated for a proximal radius fracture (ICD-10 S52.1) between 2014 and 2019 were identified from our institutional fracture registry. Patient demographics (age, sex, injury type, hand dominance) were recorded. A higher-energy mechanism was defined as a fall from >1 m. Fractures were classified according to Judet’s classification^5,11–13^ (Figure 1) and the Salter-Harris physeal fracture classification.^14^ Concomitant fractures were documented. Treatment details, including closed or open reduction and any internal fixation, were recorded. All surgeries were performed by, or under the supervision of, senior pediatric hand or orthopedic surgeons. During the study period, a total of 25 surgeons including 11 senior surgeons (2 pediatric hand surgeons and 9 pediatric orthopedic surgeons) were involved. No standardized treatment protocol was in place. Management generally proceeded in a stepwise manner from less invasive to more invasive interventions, beginning with closed reduction, followed by percutaneous reduction, and, if necessary, open reduction. The decision to use internal fixation was made at the discretion of the treating surgeon. Based on the initial management strategy, patients were categorized into two treatment groups: closed treatment (immobilization alone or closed reduction with cast) and surgical treatment (closed reduction with internal fixation, or percutaneous or open reduction with or without internal fixation). All radiographs were independently reviewed by a pediatric hand surgeon (K.Z.) and a pediatric radiologist (R.K.) using standardized protocols to reduce inter-observer variability. Formal inter- or intra-rater reliability analyses were not conducted. K.Z. did not participate in primary treatment of the patients. Maximum displacement (mm) and angulation (°) were measured on Anteroposterior and lateral views; the larger value was chosen for further statistical analyses (Figure 2). Radiographic assessments were conducted before the evaluation of final clinical outcomes. All follow-up radiographs were independently reviewed by a pediatric radiologist (R.K.) who was not involved in outcome assessment. Thus, radiographic assessment was independent of clinical outcome evaluation, although full blinding was not feasible in this retrospective study. Complications were documented for the entire cohort and classified as fracture-related or iatrogenic.

### Outcomes

**Figure 3.**
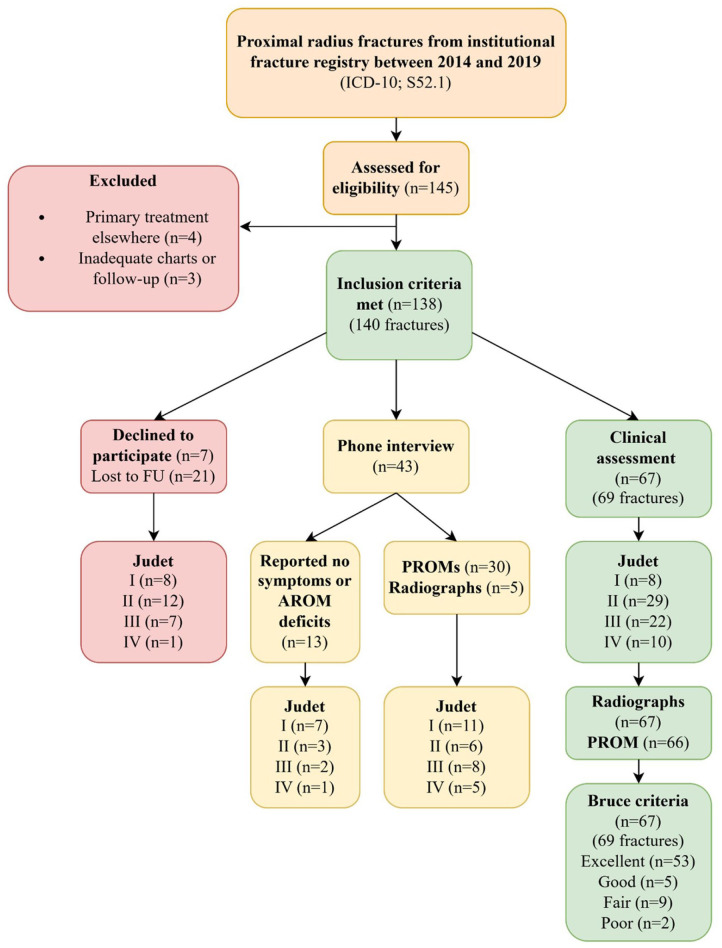
Flowchart showing patient inclusion and exclusion criteria, follow-up participation, and fracture distribution by Judet classification, PROM, and outcome data according to Bruce et al.^17^ PROM: patient-reported outcome measures

All patients were invited to a clinical follow-up ≥5 years after injury, conducted either in person or by telephone. In-clinic assessment included elbow and forearm active range-of-motion (ROM) ratios (injured vs. contralateral) using a goniometer, evaluation of elbow stability (valgus and chair tests),^15^ assessment of distal radioulnar joint (DRUJ) stability and ulnar wrist pain,^16^ and grip strength measurement with a Jamar dynamometer (Lafayette Instrument Company, Lafayette, IN, USA). The Bruce classification, originally introduced for Monteggia fractures, was applied to patients attending the clinic (Table 1).^17^ PROM comprised the Disabilities of the Arm, Shoulder and Hand (QuickDASH),^18–20^ the Pediatric Quality of Life Inventory (PedsQL 4.0 generic core scales) core and Pain modules, and cosmetic satisfaction on a 0–10 visual analog scale.^21,22^ Patients who declined an in-clinic follow-up visit were contacted by telephone, and PROMs were completed during the phone interview. Thirteen patients declined to complete the PROMs but reported overall recovery of the upper extremity. ROM could not be objectively assessed during telephone interviews; therefore, the Bruce classification could not be determined for patients assessed by phone. Categorization according to the Judet classification is presented in Figure 3. Data from patients who did not participate in the follow-up were also reviewed in accordance with the study approval. Functional outcomes could not be assessed in these patients; however, complications were recorded.

**Table 1.** Criteria for outcomes originally reporting Monteggia fractures, adapted from Bruce et al.^17^

Range of motion (60 points)
Number of points of ROM = 60—(percent impairment of upper extremity × 0.6)
ADL and work status (20 points)
20—Function equal to opposite arm
15—Independent ADL; no more than two work handicaps
10—Unable to do more than three ADL; three or more work handicaps; occupational change required
5—Unable to do four or more ADL; occupational disability
Pain (15 points)
15—No pain
13—Annoying pain with no compromise of activity
10—Pain interfering with activity
5—Pain prevents some activity
0—Pain-causing outcries and preventing activities
Anatomy (5 points)
1—Acceptable cosmetic appearance
1—No clinical angulation
1—No Clinical displacement
1—Clinical change of carrying angle less than 10 degrees
1—Roentgenographic union
Results
Excellent: 96–100; Good: 91–95; Fair: 81–90; Poor: Below 80

ADL: Activities of daily living.

### Statistics

We considered excellent (96–100 points) and good (91–95 points) Bruce categories as successful and points below 91 as an unsuccessful outcome in the statistical analyses. The groups were dichotomized into two major categories, as a categorical variable was deemed statistically more robust in this setting. We described the data using medians and interquartile ranges (IQRs) along with counts and percentages. We compared the complication groups and successful outcomes using Mann–Whitney U tests and Fisher’s tests. We visually assessed the normality of the variables and decided to use non-parametric tests. We also compared the three treatment groups: (1) closed treatment (cast immobilization or closed reduction and cast), (2) percutaneous, and (3) open reduction (with/without internal fixation) using Kruskal–Wallis tests and Fisher’s tests. To further investigate the outcomes, we fitted logistic regression models for both complications and successful outcomes. Holm’s method for correction was used. We considered *p*-values under 0.05 to be significant. Kaplan–Meier analysis was used to assess the distribution of unsuccessful functional outcomes over follow-up time. An unsuccessful outcome was assessed at final follow-up, Kaplan–Meier analysis reflects cumulative incidence across follow-up duration rather than exact time to functional deterioration. We visualized the risk for any complication using locally estimated scatterplot smoothing (LOESS)^23^ for select variables. We winsorized the variables to 2.5% and 97.5% to avoid long uninformative tails in the plots. Winsorization was applied by replacing extreme displacement values beyond predefined percentiles with boundary values. All analyses were done using R software version 4.5.1. (R Core Team (2025). R: A Language and Environment for Statistical Computing. R Foundation for Statistical Computing, Vienna, Austria. <https://www.R-project.org/>.)

## Results

### Fracture demographics

A total of 138 children (70 girls) with 140 proximal radius fractures were included (no open fractures). The median age at injury was 9.8 years (range 1–16, IQR 8–11). Higher energy mechanisms were associated with greater deformity when compared with low energy mechanisms, but the clinical relevance of this difference was limited (median angulation 31° vs. 21°, displacement 4 mm vs. 1.5 mm; both *p* = 0.029). Most physeal fractures were Salter-Harris type II and only one type IV. One intra-articular fracture was noted with a closed physis. According to Judet’s classification, most fractures were mild to moderate (grades I–II, 60%), while 40% were severe (grades III–IV). Concomitant injuries included 40 Monteggia variants, 1 Bado type IV Monteggia-, 6 medial humeral epicondyle-, 2 lateral humeral epicondyle-, 1 forearm shaft- and 1 distal forearm fractures (Table 2).

**Table 2.** Fracture demographics.

	Judet 1	Judet 2	Judet 3	Judet 4	Total
	(*N* = 34)	(*N* = 50)	(*N* = 39)	(*N* = 17)	(*N* = 140)
Sex (%)					
Male	23 (68)	24 (48)	15 (38)	7 (41)	69 (49)
Female	11 (32)	26 (52)	24 (62)	10 (59)	71 (51)
Median age at injury	11	9	9	10	10
(IQR) [range]	(9–13) [4–16]	(6–10) [1–14]	(7–10) [4–13]	(8–11) [8–15]	(8–11) [1–16]
Fracture type (%)					
Metaphyseal fracture	25 (74)	19 (38)	8 (21)	1 (6)	53 (38)
Physeal fracture	9 (26)	31 (64)	31 (79)	16 (94)	87 (62)
Injury mechanism (%)					
High energy	6 (18)	31 (62)	27 (69)	11 (65)	75 (54)
Low energy	28 (82)	19 (28)	12 (31)	6 (35)	65 (46)
Median primary angulation	15	21	39	71	26
(IQR) [range]	(13–17) [5–22]	(18–26) [7–35]	(36–46) [31–59]	(63–79) [20–108]	(17–39) [5–108]
Median primary displacement	0	2	5	14	3
(IQR) [range]	(0) [0–2]	(1–3) [0–5]	(4–6) [4–10]	(13–15) [11–16]	(1–5) [0–16]
Concomitant fractures (%)	9 (26)	23 (46)	16 (41)	5 (29)	53 (38)
Treatment (%)					
Immobilization without reduction	34 (100)	42 (84)	2 (5)		78 (56)
Closed reduction and cast		4 (8)	9 (23)	1 (6)	14 (10)
Percutaneous reduction and cast		1 (2)	3 (8)	1 (6)	5 (4)
Open reduction and cast			1 (3)	1 (6)	2 (1)
Closed reduction and internal fixation		3 (6)	12 (31)	3 (18)	18 (13)
Percutaneous reduction and internal fixation			7 (18)	7 (41)	14 (10)
Open reduction and internal fixation			5 (13)	4 (24)	9 (6)
Median follow-up time	7.2	6.5	7.2	6.5	6.8
(IQR) [range]	(5.7–8.2) [5.2–9]	(5.7–7.5) [5–9.7]	(5.8–8) [5–9.7]	(5.3–8) [5.2–9]	(5.6–8) [5–9.7]

IQR: interquartile range. Age and follow-up time in years.

### Received treatment

The choice of treatment was determined by the surgeon, with the median angulation of 18° (range 5–42, IQR 14–22) and displacement of 1 mm (range 0–5, IQR 0–2) in the 78 unreduced fractures, compared to 33° (range 18–53, IQR 27–37) and 4 mm (range 1–6, IQR 4–5) in 14 fractures treated by closed reduction and 46° (range 2–108, IQR 37–63) and 6 mm (range 0–15, IQR 4–12) in 48 fractures that were treated surgically (open or percutaneous reduction without internal fixation (*n* = 7), closed reduction and internal fixation (*n* = 18) and open or percutaneous reduction with internal fixation (*n* = 23)). Internal fixation included mostly intramedullary nail (*n* = 37), followed by biodegradable implants (*n* = 3) and locking plate (*n* = 1). Seven concomitant olecranon and one ulnar shaft fractures were internally fixed. Independent predictors of open reduction were greater initial fracture angulation and displacement, physeal involvement (*p* < 0.001), and treatment delay of ≥3 days (*p* = 0.005).

### Complications

Thirty-five (25%) of the 138 children had complications, of which 30 occurred in children with a physeal fracture. The most common complication, premature physeal closure, developed in 15 (11%) children (Table 3, Supplemental Table).

**Table 3.** Univariate and multivariate regression models on complications.

Model	Term	OR	*p* value
Univariate			
Age at injury	1 year increase	1.00 (0.88−1.15)	0.970
Displacement ≥3 mm	Yes	6.70 (2.72−19.12)	**0.001**
Physeal fracture	Yes	5.05 (1.96−15.72)	**0.011**
Concomitant ulnar fracture	Yes	2.28 (1.01−5.10)	0.133
Treatment delay ≥3 days	Yes	3.69 (1.32−10.41)	0.055
Judet’s class (no class 1)	3 (vs. 2)	1.58 (0.62−4.06)	0.665
	4 (vs. 2)	4.52 (1.44−15.11)	**0.055**
Judet’s class comparison	3–4 (vs. 1–2)	4.18 (1.89−9.66)	**0.004**
Multivariate			
Judet’s class and displacement	Judet’s class: 3 (vs. 2)	0.92 (0.28−3.17)	0.887
	Judet’s class: 4 (vs. 2)	2.62 (0.67−11.05)	0.521
	Displacement ≥3 mm: yes	2.45 (0.64−9.57)	0.521
Age, physeal fracture, and ulnar fracture	Age at injury (1yr increase)	1.05 (0.89−1.23)	0.579
	Physeal fracture: yes	4.57 (1.75−14.34)	**0.012**
	Concomitant ulnar fracture: yes	2.02 (0.83−4.93)	0.237
Physeal fracture, displacement, and ulnar fracture	Physeal fracture: yes	2.46 (0.91−7.43)	0.156
	Displacement ≥3 mm: yes	5.05 (1.94−14.99)	**0.006**
	Concomitant ulnar fracture: yes	2.03 (0.84−4.90)	0.156
Physeal fracture, Judet’s class, and ulna fracture	Physeal fracture: yes	2.56 (0.94−7.78)	0.116
	Judet’s class: 3–4 (vs. 1–2)	3.27 (1.36−8.20)	**0.030**
	Concomitant ulnar fracture: yes	2.28 (0.95−5.54)	0.116

The sample size did not allow for any more terms in the model. To explore the potential effects between the explanatory variables, we decided to fit multiple models. This was planned post hoc. *p*-Value corrections were performed by Holm’s method. Significant *p* values are bolded.

**Figure 4.**
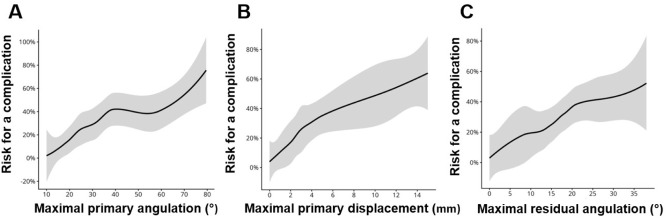
LOESS risk plots showing the estimated risk of any complications for maximal primary angulation (°) (a), maximal primary displacement (mm) (b) and maximal residual angulation (°) (c). All of them show a trend of risk increasing risk of complications with higher values. The black line represents the point estimate and the shaded area the 95% confidence interval. Values were winsorized to the 2.5 and 97.5 percentiles to avoid uninformative tails. LOESS: locally estimated scatterplot smoothing.

The rate of complications in children with Judet type I fractures was 0% (0/34), Judet II 24% (12/50), Judet III 33% (13/39), and Judet type IV 59% (10/17). Higher Judet class, displacement ≥3 mm, and physeal involvement (all *p* < 0.001) predicted complications. The rate of complications was higher in children who had surgery (21/48 vs. 14/92, *p* = 0.001) or treatment delay ≥3 days (9/18 vs. 26/122, *p* = 0.02). In the univariate regression analysis, after the correction with Holm’s method, displacement ≥3 mm (odds ratio [OR] 6.7, 95% confidence interval (CI) 2.7–19.1), physeal fracture (OR 5.1, 95% CI 2.0–15.7), and higher Judet classification (III–IV vs. I–II; OR 4.2, 95% CI 1.9–9.7) were significantly associated with complications. In the multivariate model, only physeal fracture, displacement, and higher Judet class remained independent risk factors (Table 3). Children with complications had their fractures immobilized in poorer alignment compared to children without complications, but this was not statistically significant (median angulation 20° vs. 17°, displacement 2 mm vs. 1 mm, *p* = 0.09 and *p* = 0.6). A threshold in the cumulative complication risk was observed at 3–4 mm of initial displacement (Figure 4).

### Treatment outcomes

At a median of 6.8 years (range 5.6–8, IQR 7–8), 110/138 (80%) patients (2 bilateral) were assessed: 67 (69 fractures, 2 bilateral) attended clinical and radiographic follow-up, and 30 completed PROMs via telephone interview. In addition, 13 patients reported no symptoms or functional impairment of the upper limb (Figure 3). An unsatisfactory Bruce outcome was recorded in 11/67 (16%) patients, and the rates by Judet’s classification were as follows: I 0/8, II 3/29 (10%), III 4/22 (18%), and IV 4/10 (40%). Nine of these patients experienced a complication. PROMs indicated generally favorable outcomes (Table 4). Four of 43 patients interviewed by telephone had previously documented complications. Objective Bruce classification could not be assessed; however, median QuickDASH was 0 (range 0–9.1; IQR 0–0), median PedsQL total score 94 (range 72–100; IQR 100–100), and median PedsQL physical score 94 (range 75–100; IQR 100–100).

**Table 4.** Successful outcome = excellent or good result according to the Bruce scoring system.^17^

Variable median (IQR)	All (*n* = 67)	Successful outcome (*n* = 56)	Unsuccessful outcome (*n* = 11)	*p* value	Difference CI 95%
AROM	0 (0−5)	0 (0−5)	28 (14−49)	0.000	25 (15, 40)
Pain worst	0.0 (0.0−1.0)	0.0 (0.0−0.3)	2.2 (2.0−4.1)	0.000	2.1 (1.9, 3.3)
PedsQL	90.2 (80.4−96.8)	91.3 (82.0−97.8)	75.0 (67.4−78.0)	0.001	−16.3 (−22.8, −7.6)
PedsQL PF	93.5 (85.2−100.0)	93.8 (87.5−100.0)	82.8 (68.7−89.8)	0.004	−12.2 (−18.8, −3.2)
Cosmetic VAS	10.0 (8.0−10.0)	10.0 (8.3−10.0)	7.9 (7.5−9.1)	0.014	−1.3 (−2.0, −0.0)
QuickDASH	0.0 (0.0−2.3)	0.0 (0.0−1.7)	11.7 (5.7−18.8)	0.000	11.7 (5.3. 15.0)

Unsuccessful outcome = fair or poor result according to the Bruce scoring system. CI: 95% confidence interval, showing the lower and upper limits. *p*-Values were calculated using the Mann–Whitney U test between successful and unsuccessful outcomes. *p*-Value corrections were performed by Holm’s method. AROM: active range of motion total difference; PF: Physical functioning; VAS: visual analog scale.

Among 61 Judet II–IV cases, median residual angulation was 19° (range 0–45, IQR 14–25) and displacement 2 mm (range 0–12, IQR 0–2), with no difference between unsatisfactory (*n* = 11) and satisfactory (*n* = 50) Bruce outcomes. Stratification by age (<10 years vs. ≥10 years) revealed no statistically significant differences between successful and unsuccessful outcomes in either age group (<10 years: 20° vs. 19°, 2 mm vs. 2 mm; ≥10 years: 16° vs. 22°, 1 mm vs. 0 mm).

Elbow valgus deformity with >10 ° asymmetry in carrying angle was observed in 12% (8/67), 4 with poor outcomes. Laxity of the elbow was recorded in 10 children, and DRUJ instability in 2 patients. Three patients with elbow laxity and one patient with DRUJ instability had unsuccessful functional outcomes according to the Bruce classification. Median grip strength 29 kg (12–64, IQR 20–36) in the affected hand and 28 kg (12–58, IQR 22–35) in the non-affected hand were observed, with no correlation to Bruce outcomes.

Children with unsatisfactory outcomes had significantly poorer ROM, higher QuickDASH and pain scores, lower PedsQL total and physical functioning scores, and reduced cosmetic satisfaction compared with those with satisfactory outcomes (Table 4). Kaplan–Meier analysis demonstrated that unsuccessful outcomes were evenly distributed over follow-up, with no evidence of late functional deterioration. Unsuccessful outcomes were associated with surgical treatment (7/22 vs. 4/47, OR 5.0, 95% CI 1.3–19.6), need for reoperation (3/4 vs. 8/65, OR 21.4, 95% CI 2.0–231.2), and the occurrence of complications (9/28 vs. 2/41, OR 9.2, 95% CI 1.8–47.0). In regression analysis, surgical treatment (OR 5.0, 95% CI 1.3–21.5) was the only independent predictor of unsuccessful outcome, although this finding is likely influenced by confounding by indication, as surgically treated fractures were more severe.

## Discussion

Of 140 pediatric proximal radius fractures, 110 patients had a minimum follow-up of 5 years. Displacement >3 mm, severe angulation, and physeal involvement were associated with complications, occurring in approximately one-fourth of cases, and 16% of patients had unfavorable outcomes. This study represents one of the larger cohorts with medium-term outcome data for these injuries. The Judet classification is widely used for pediatric proximal radius fractures but has been criticized for limited reproducibility due to variation in radiographic assessment of angulation, as Wang et al.^24^ recently showed in their comprehensive comparison of plain radiographs and computed tomography scans. Despite these limitations, we applied this classification because of its broad acceptance in the literature. The original classification considered only angulation,^11^ but displacement was later incorporated.^12^ In this study, displacement was measured in millimeters rather than as a percentage, as we believe that absolute displacement combined with angulation provides more clinically meaningful information than classification alone. Nonetheless, measurement error at the millimeter level is possible due to variations in the imaging angle and technical factors such as detector positioning. We identified a complication threshold between 3 and 4 mm, estimated using LOESS smoothing with winsorization of displacement values to limit the influence of extreme outliers. This justified the use of 3 mm as the displacement cut-off in our statistical analyses.

Undisplaced fractures were also included, explaining the high proportion (56%) of cases managed with immobilization alone, which is consistent with Schmittenbecher’s report of 55%.^9^ Among displaced fractures, 28% underwent open or percutaneous reduction, which is comparable to the findings of Zimmerman et al.,^7^ who reported surgical treatment of 151 displaced fractures in 2014. Their reoperation rate was 7%, whereas our study demonstrated a lower rate of 2%.

Complications were observed in 25% of fractures, which is in line with earlier literature.^1,7^ While not all complications led to unsuccessful outcomes, they were strongly associated with them. Of these, 9 of 35 (26%) were treatment-related, most often due to inadequate primary reduction. Prior studies suggest that unstable fractures require careful management, and that internal fixation should be considered whenever open reduction is performed.^25^ In our series, 7 of 30 openly reduced fractures were not internally fixed. None required re-reduction, and only one developed growth arrest, which did not affect the outcome. However, given the small number of cases, no firm conclusions can be drawn regarding the need for routine internal fixation after open reduction.

Physeal fractures accounted for 62% of cases in our series. We found that physeal fractures were highly represented in patients with complications. Growth arrest is frequently reported and rarely affects the outcome.^5,7^ Premature physeal closure occurred in 17% of physeal fractures in our cohort, resulting in poor outcomes in 2 of the 10 cases for which follow-up data were available. Growth disturbance was not the sole contributor to poor outcomes, as malunion and valgus deformity were also present in these cases. Therefore, although premature physeal closure was common, the small number of cases with available follow-up limits conclusions regarding its clinical impact on long-term outcomes.

Nerve injuries were rare: three cases (2%), two of these fracture-related, with only one permanent deficit. One superficial radial nerve lesion was associated with intramedullary nail entry, also a known risk factor.^26^

We found that complications were primarily associated with injury-related factors, including initial displacement, angulation, and physeal involvement. Treatment delay ≥3 days appeared to be the only modifiable factor associated with complications in the unadjusted analysis. However, this association was not maintained after adjustment. Zimmerman et al.^7^ similarly identified time to surgery as a risk factor, although in their cohort shorter time to surgery was associated with poorer outcomes. These findings are conceptually related and likely reflect confounding by injury severity, as more severe fractures are typically treated earlier.^10^ Therefore, the true impact of treatment delay remains uncertain, although our data suggest that prolonged delay may increase complication risk. Other factors, such as surgeon-related variables and timing of surgery, may also influence complications but could not be assessed in this study. Although open reduction appeared to increase the risk of complications, this association is likely confounded by the fact that more severely displaced fractures are more often managed operatively. In multivariate analysis, only fracture-related variables, mainly angulation, displacement, and physeal involvement, remained significantly associated with complications.

For long-term outcome assessment, we aimed to use a classification sensitive enough to detect poor results. Previous studies have used complications and ROM loss,^7^ the Tibone classification,^1^ the Mayo Elbow Score^27^ or the Metaizeu functional scoring.^28,29^ We chose the Bruce classification, originally developed for Monteggia fractures,^17^ as it incorporates multiple functional domains while emphasizing motion deficits. Using this system, we found a 16% risk of poor long-term outcome, which is lower than reported by Basmajian and Zimmerman^1,7^ and comparable to Schmittenbecher et al.^9^

We also evaluated patient-reported outcomes. Overall, QuickDASH and PedsQL scores were similar to normative population scores,^18^ but significantly worse in children with unsuccessful outcomes. It is noted that although there is limited evidence to support the use of QuickDASH in pediatric orthopedic and fracture settings, it might exhibit a ceiling effect in pediatric populations with upper-extremity fractures.^19,20^ Similarly, as PedsQL represents a more generic, not upper-extremity-specific PROM, it may be unable to detect minor dysfunction after proximal radius fractures. In our view, objective measures such as the Bruce classification, therefore, provide more reliable outcome assessment than the PROMs currently available.

Previous studies have identified older age, primary displacement, short time to surgery^7^ and open reduction as risk factors for poor outcomes.^7,9,27,29^ We could not identify older age as a risk factor for either complications or unsuccessful outcomes which deviates from the earlier reports. Although Kaplan–Meier analysis showed no evidence of late functional deterioration, the small number of unsuccessful outcomes limits the statistical power to detect time-dependent differences.

Langenberg et al.^30^ reported in a recent systematic review that open reduction was associated with poor functional outcomes in approximately half of patients, whereas fractures managed with closed reduction had favorable outcomes in more than 90% of cases. We found a correlation between treatment type (open vs. closed) and outcome, but as more severely displaced fractures generally required more invasive interventions, this created selection bias. Although our results suggest that reoperation is associated with worse outcomes, the small number of patients requiring revision surgery (*n* = 4) weakens the statistical power of this finding. Similarly, while post-treatment angulation was slightly higher in the complication group, the differences were small. After adjustment for age, the association between post-treatment angulation and complications was not strengthened, and the small sample size limits conclusions regarding its clinical significance. Together with the lack of differences in outcomes related to residual deformity, these findings raise questions regarding the clinical relevance of residual deformity after treatment.

The findings of this study are largely consistent with previous literature. Our results add to existing knowledge by showing that complications were more frequent in fractures with displacement ≥3 mm. However, we were unable to determine whether these fractures should be managed more aggressively with surgical treatment, and further studies are needed to clarify optimal treatment thresholds. In contrast to some previous studies, we observed a slightly higher risk of complications in fractures with treatment delay >3 days, although this was not statistically significant.

## Strengths and limitations

The major strengths of this study are the long-term follow-up of nearly 7 years, the high follow-up rate, and the inclusion of both PROMs and functional outcome measures. This was a retrospective study, and treatment decisions regarding open reduction and internal fixation were made at the discretion of the surgeon, introducing inter-surgeon variability. Despite a relatively large cohort, the number of poor outcomes was small, limiting statistical power and making it difficult to reliably identify all risk factors. Follow-up assessments were heterogeneous, as some patients were evaluated clinically and others by telephone, which may have influenced outcome assessment. In addition, approximately 20% of patients were lost to follow-up, introducing potential attrition bias. Finally, this was a single-center study, which may limit the generalizability of the findings.

## Conclusion

In this retrospective cohort of pediatric proximal radius fractures, greater fracture angulation, primary displacement ≥3 mm, and physeal involvement were associated with an increased risk of complications, which, in turn, were linked to unfavorable functional outcomes at a minimum 5-year follow-up.

## Supplemental Material

sj-docx-2-cho-10.1177_18632521261434093 – Supplemental material for Primary displacement predicts complications and poorer outcomes after pediatric proximal radius fractures: A retrospective study of 140 fractures Supplemental material, sj-docx-2-cho-10.1177_18632521261434093 for Primary displacement predicts complications and poorer outcomes after pediatric proximal radius fractures: A retrospective study of 140 fractures by Kaj Zilliacus, Yrjänä Nietosvaara, Ilkka Helenius, Reetta Kivisaari, Niko Kämppä and Petra Grahn in Journal of Children's Orthopaedics

sj-pdf-1-cho-10.1177_18632521261434093 – Supplemental material for Primary displacement predicts complications and poorer outcomes after pediatric proximal radius fractures: A retrospective study of 140 fractures Supplemental material, sj-pdf-1-cho-10.1177_18632521261434093 for Primary displacement predicts complications and poorer outcomes after pediatric proximal radius fractures: A retrospective study of 140 fractures by Kaj Zilliacus, Yrjänä Nietosvaara, Ilkka Helenius, Reetta Kivisaari, Niko Kämppä and Petra Grahn in Journal of Children's Orthopaedics
