# Supplementary material for: Primary displacement predicts complications and poorer outcomes after pediatric proximal radius fractures: A retrospective study of 140 fractures
Source: J Child Orthop. 2026 Mar 25;20(3):266–76. doi: 10.1177/18632521261434093 (PMC13017641; doi:10.1177/18632521261434093)
Supplement: sj-docx-2-cho-10.1177_18632521261434093 – Supplemental material for Primary displacement predicts complications and poorer outcomes after pediatric proximal radius fractures: A retrospective study of 140 fractures [file sj-docx-2-cho-10.1177_18632521261434093.docx]

Table 3. Supplementary Table. Patient complications stratified by Judet fracture type, age, and treatment modality. Treatment-related complications are marked with an asterisk (*). CI = long-arm cast immobilization; CR = closed reduction; OR = open reduction; FIN = flexible intramedullary nailing (Métaizeau technique¹²); valgus = valgus deformity; HO = heterotopic ossification; PA = physeal arrest; RHJ = radiohumeral joint; NI = nerve injury; LoR = loss of reduction; AVN = avascular necrosis.

| **Fracture Type (Judet)** | **Age at injury (years)** | **Treatment Primary / Secondary** | **Complication** | **Explanation for iatrogenic complication** |  |
| --- | --- | --- | --- | --- | --- |
|  |  |  |  |  |  |
|  |  |  |  |  |  |
| II | 5 | CR | Valgus, PA |  |  |
| II | 5 | CI | PA |  |  |
| II | 6 | CI | Valgus |  |  |
| II | 7 | CI | Valgus |  |  |
| II | 8 | CI | Valgus, HO |  |  |
| II | 8 | CR | PA |  |  |
| II | 8 | CI | PA |  |  |
| II | 8 | CI | Valgus* | Insufficient CR |  |
| II | 10 | CI | Valgus, NI (ulnar) |  |  |
| II | 10 | CI | HO, PA, incongruent RHJ |  |  |
| II | 10 | CI | Valgus, megacaput |  |  |
| II | 14 | CI | HO |  |  |
| III | 7 | OR with FIN | HO* | Insufficient OR |  |
| III | 8 | CR / OR with K-wires | HO* | Insufficient CR |  |
| III | 8 | OR with FIN | NI (radial)* | FIN entry point |  |
| III | 8 | CI / physeal bar resection / CO | PA, incongruent RHJ* | Insufficient CR |  |
| III | 8 | OR with FIN | Incongruent RHJ |  |  |
| III | 9 | OR with FIN | PA |  |  |
| III | 9 | CR with FIN | PA, incongruent RHJ |  |  |
| III | 9 | OR with FIN | PA |  |  |
| III | 10 | CR | Incongruent RHJ, megacaput |  |  |
| III | 11 | CR with FIN | NI (radial) |  |  |
| III | 12 | OR | PA |  |  |
| III | 12 | CR / OR with FIN | LoR* |  |  |
| III | 13 | CR / OR with K-wires | LoR*/HO | Inssufficient CR |  |
| IV | 9 | CR with FIN | HO |  |  |
| IV | 8 | OR with FIN | PA |  |  |
| IV | 8 | OR with Bio-pins | HO, Incongruent RHJ |  |  |
| IV | 10 | OR with FIN | PA, tendon injury (EPL)* | FIN entry point |  |
| IV | 10 | CR with FIN | PA |  |  |
| IV | 10 | CR with FIN | PA |  |  |
| IV | 11 | OR / OR with K-wires | LoR* / AVN | Insufficient OR |  |
| IV | 11 | OR with FIN | AVN |  |  |
| IV | 11 | OR with FIN | PA, megacaput |  |  |
| IV | 15 | OR with FIN | Valgus |  |  |
